# English language proficiency, complete tooth loss, and recent dental visits among older adults in the United States

**DOI:** 10.1177/2050312120962995

**Published:** 2020-10-07

**Authors:** Andriana M Foiles Sifuentes, Maira A Castaneda-Avila, Kate L Lapane

**Affiliations:** Department of Population and Quantitative Health Sciences, University of Massachusetts Medical School, Worcester, MA, USA

**Keywords:** limited English proficiency, Spanish, edentulism, complete tooth loss

## Abstract

**Objectives:**

This study sought to provide population-based estimates of complete tooth loss and recent dental visits among older adults in the United States by English language proficiency.

**Methods:**

We conducted a cross-sectional analysis of the 2017 Medical Expenditure Panel Survey among participants ⩾50 years of age (n = 10,452, weighted to represent 111,895,290 persons). Five categories of language proficiency were created based on self-reported English language ability and language spoken at home (Spanish, Other).

**Results:**

The prevalence of complete tooth loss was higher among those with limited English proficiency (Spanish speaking: 13.7%; Other languages: 16.9%) than those proficient in English (Spanish speaking: 5.0%; Other languages: 6.0%, English only: 12.0%). Complete tooth loss was less common among participants for whom Spanish was their primary language, with limited English proficiency relative to English only (adjusted odds ratio: 0.56; 95% confidence interval: 0.42–0.76). Among those without complete tooth loss, dental visits in the past year were less common among participants with primary language other than English as compared to those who only speak English.

**Conclusions:**

Complete tooth loss varied by English language proficiency among adults aged ⩾50 years in the United States. Suboptimal adherence to annual dental visits was common, more so in those with complete tooth loss, and varied by English language proficiency.

## Introduction

In the United States, people with limited English proficiency (LEP), for whom English is a second language, and those who possess limited function of reading, writing, or speaking English have decreased access to health care and related services.^1^ Lack of access to oral health care services has been noted for persons with LEP, particularly among aging populations in the United States.^2^ LEP adversely affects access to dental care, and lacking teeth among older populations serves as a proxy for dental care access across the life course.^3^

Dental providers report feeling underprepared to care for persons with LEP in the United States.^4^ Unfortunately, not all safety net dental clinics recognize that in the United States, there is a legal obligation to care for persons with LEP.^5^ For aging persons with LEP in the United States, access to dental care is a pressing concern given the relationship of biological aging with oral health decline.^6^ Oral health is linked to behavioral and social factors, and persons from vulnerable communities are at higher risk of oral disease and tooth loss.^2,7^ Research examining oral health among aging persons with LEP is scant. Globally, populations are aging rapidly, making the intersection of oral health, aging, and persons with LEP a critical focal point.^8^ Furthermore, persons with LEP in the United States come from many countries. Targeted interventions to improve dental access must consider different languages to be successful.^9^ Because the population of aging persons with LEP is growing in the United States,^10^ research on this topic is sorely needed.

Using nationally representative data, this study sought to provide population-based estimates of complete tooth loss by LEP status among older adults in the United States. We hypothesized that we would observe greater prevalence of complete tooth loss among non-English speaking adults in the United States. The study also sought to estimate the proportion of older adults in the United States who had a recent dental visit by LEP status.

## Methods

The Westat Institutional Review Board by the Office for Protection from Research Risk approved the original study design.^11^ Written informed consent was received by Westat, who ran the study. Because the data for this study were de-identified, anonymized, and released as publicly available data, the University of Massachusetts Medical School Institutional Review Board deemed the study did not need to go through ethics review. Therefore, we do not have a waiver number to include.

### Study design

We conducted a cross-sectional study.

### Data source

Data were drawn from the 2017 Medical Expenditure Panel Survey (MEPS), a nationally representative sample of non-institutionalized civilians in the United States (see online Supplemental Material). The Agency for Healthcare Research and Quality and the Centers for Disease Control sponsored the data collection for MEPS 2017. Persons were randomly selected to participate in the household survey. Based on responses to the questionnaires, the participant’s medical and dental providers may have been sent questionnaires.^11^ MEPS data can be analyzed for individual person-level responses.^12^

### Study sample

The MEPS 2017 Household Component had 31,880 participants. We used the Household Component because this part of the survey had the information needed to evaluate our study purpose.^11^ We excluded participants who were <50 years of age and those with “don’t know” responses, those with missing data, or those who refused to respond to questions regarding tooth loss, language proficiency, education, marital status, years in the United States, or born in the United States. The remaining 10,452 respondents comprised our sample (weighted to be representative of 111,895,290 civilians in the United States).

### Operational definition of LEP

Participants were grouped into five categories based on two variables: (1) their self-reported English language ability (LEP, proficient) and (2). language spoken at home (Spanish, Other). In the MEPS Household Component, respondents were asked by interviewers: “How well {do/does} {you/person} speak English? Would you say . . . Very well; well; Not well; Not at all?.”^1^ We first categorized participants as (1) having LEP (not well; not at all); English proficient (well; very well) and English only (two-thirds reported the question as not pertaining to them).^13^ We then used the MEPS Household Component survey question: “What language do you speak at home? Would you say . . . English, Spanish, Other” to differentiate primary language. Five categories were created. Those with LEP who reported speaking Spanish at home were categorized as Spanish speaking, with LEP (SLEP). Those with LEP who reported speaking another language at home were categorized as Other language, with LEP (OLEP). English-proficient respondents were classified as Spanish speaking (SEP), English (only language spoken, referent group), or other language (OEP).

### Operational definitions of outcome variables

Two outcomes were of interest. The first outcome was self-reported complete tooth loss (all teeth from upper and lower jawbone). Participants were asked: “Have you . . . lost all upper and lower teeth?” (Yes/No). The second outcome variable of interest was whether or not participants reported a recent dental visit. The MEPS survey included the following question: “How many dental visits [. . . did you have . . .] in the last 12 months?.” We recoded this variable as any visits versus no visits.

### Covariates

We included demographic variables that could affect an individual’s ability to access dental services. These included age, sex, race (Asian, Black, White), marital status (married, divorced/widowed/separated/never married), education (no degree, high school diploma (or equivalent), some college or beyond), and family income as percentage of poverty line (poor/negative, near poor, low income, middle income, high income). We created a variable to describe number of years living in the United States by combining information from two questions: (1) “Were you born in the United States (yes/no)”? and (2) “In what year did you come to the United States to stay?.” Using this information and the respondent’s age, we categorized participants as born in the United States, <15 years in the United States, or ⩾15 years in the United States. We selected 15 years as the cut point based on the distribution of the variable in our sample. We included variables for insurance coverage (private, public (Medicare or Medicaid), uninsured), dental insurance coverage (yes/no), and smoker in the last 12 months (yes/no).

### Data analysis

To derive population-based estimates, we followed the MEPS recommendations for data analysis.^14^ We described the characteristics of the population by complete tooth loss. For continuous variables, means and standard deviations were calculated. Percentages were calculated for categorical variables. We considered differences in the characteristics by complete tooth loss of at least 5% to be clinically relevant. Then, we conducted an analysis stratified by English language proficiency. Using logistic models, we quantified the association between English language proficiency and complete tooth loss. The outcome variable was complete tooth loss. The primary determinant was English language proficiency (Spanish LEP, Other LEP, Spanish-English proficient, Other-English proficient, English only (reference group)). Before including the covariates described above in the model, we evaluated the potential for multicollinearity by calculating correlations between the variables. None were highly correlated (>0.80). To understand the role of education (a proxy of socioeconomic status in childhood and adolescence) and current family income (a proxy for current socioeconomic status), we built a series of models. First, we included language proficiency, age, sex, marital status, smoking, and dental insurance. Then, we added family income to the model. Next, we added education (but not family income). Finally, we estimated a full model with language proficiency, age, sex, marital status, smoking, dental insurance, education, and family income. From each model, we derived adjusted odds ratios (aOR) and corresponding 95% confidence intervals (CI).

We then evaluated the association between LEP and whether or not the participant had a dental visit in the past 12 months. We used logistic models, stratified by complete tooth loss. The outcome variable was the binary indicator for whether or not the participant had a dental visit in the past 12 months. English language proficiency was included as the primary determinant of interest. We used the same approach as described above to develop crude, partially adjusted models, and a fully adjusted model. Although we realize that the analyses among those with complete tooth loss lack precision, we show results for completeness.

## Results

The overall percent of adults aged ⩾50 years in the United States with self-reported complete tooth loss of the upper and lower jaw was 11.4%. Table 1 shows that on average, those with complete tooth loss were older than those without complete tooth loss (average age: 69.8 years in those with complete tooth loss versus 63.8 years in those without). The distribution of sex and race/ethnicity was similar by edentulism status, but fewer older adults with complete tooth loss were currently married as compared to those without complete tooth loss (46.4% versus 62.0%). Sixteen percent of those with complete tooth loss were current smokers as compared to 7.0% of those without complete tooth loss. Markers of socioeconomic status including education, income, public insurance, and dental insurance all suggested that older adults with complete tooth loss had lower socioeconomic positioning than those without complete tooth loss.

**Table 1.** Characteristics of adults ⩾50 years of age with and without complete tooth loss in the United States (2017).

	Complete tooth loss	
	Yes	No
N	1353	9099
Weighted n	12,733,684	99,161,606
Mean age (years ± standard deviation)	69.8 ± 0.4	63.8 ± 0.2
Women	53.0	53.3
Race/ethnicity		
Non-Hispanic Asian	3.5	4.7
Non-Hispanic Black	12.3	10.3
Hispanic	8.0	11.2
Non-Hispanic multiracial	2.3	3.3
Non-Hispanic White	73.0	71.5
Marital status		
Married	46.4	62.0
Divorced, widowed, separated	46.7	30.4
Never married	6.8	7.6
Education		
No degree	27.4	8.9
High school diploma or equivalent	54.2	46.1
Some college or beyond	18.4	45.0
Years living in United States		
Born in United States	88.1	84.1
<15 years	2.6	1.6
⩾15 years	9.2	14.3
Family income		
Poor/negative	17.5	8.3
Near poor	7.0	3.8
Low income	22.0	10.8
Middle income	28.8	26.1
High income	24.7	51.0
Insurance coverage		
Private	42.8	70.6
Public	53.8	25.2
Uninsured	3.5	4.3
Dental insurance	15.3	39.5
Dental visit in the last year	15.7	52.2
Smoker within last year	16.5	7.0

**Figure 1.**
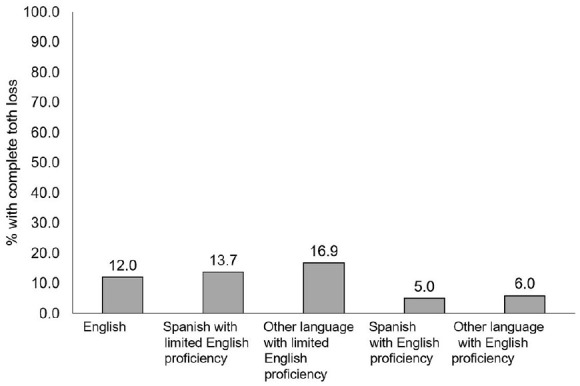
Prevalence of complete tooth loss by English language proficiency among adults aged ⩾50 years in the United States (2017).

Figure 1 shows the prevalence of complete tooth loss by language proficiency. Among those with English as a primary language, 12.0% had complete tooth loss. The prevalence of complete tooth loss was higher among those with LEP (Spanish speaking: 13.7%; other languages: 16.9%) than those proficient in English (Spanish speaking: 5.0%; other languages: 6.0%).

The characteristics of adults ⩾50 years of age with and without complete tooth loss stratified by English language proficiency is shown in Table 2. Regardless of language proficiency status, people with complete tooth loss were older and had less education, were more likely to have public health insurance, less likely to have dental insurance, and less likely to report a dental visit in the past 12 months than those without complete tooth loss. For most LEP categories, those with complete tooth loss were less likely to be married than those without complete tooth loss.

**Table 2.** Characteristics of adults ⩾50 years of age with and without complete tooth loss in the United States, stratified by English language proficiency (LEP, 2017).

Proficiency	English only		Spanish LEP		Other LEP		Spanish EP		Other EP	
Complete tooth loss	Yes	No	Yes	No	Yes	No	Yes	No	Yes	No
Unweighted n	1110	6793	119	730	26	171	63	871	35	534
Women	47.7	45.9	40.5	41.5	60.9	51.4	29.6	55.4	47.1	46.7
Mean age (years ± standard deviation)	69.7 ± 0.4	64.2 ± 0.2	72.8 ± 0.6	61.3 ± 0.3	74.4 ± 0.0	66.0 ± 0.8	67.8 ± 0.6	60.6 ± 0.4	65.9 ± 0.2	62.0 ± 0.4
Race/ethnicity										
Non-Hispanic Asian	0.2	1.1	0	0	83.2	78.9	2.6	0.7	43.1	43.6
Non-Hispanic Black	13.9	11.4	0	0.2	0	8.5	2.3	3.0	4.1	9.9
Hispanic	1.8	2.7	95.9	98.9	0	79.4	76.6	2.0	0	11.2
Non-Hispanic multiracial	3.4	2.6	0	0	0	0.7	3.4	0.9	6.7	2.2
Non-Hispanic White	80.6	82.2	4.1	0.9	16.8	10.5	15.0	16.1	46.2	42.3
Marital status										
Married	45.6	60.9	45.8	64.6	80.6	73.0	49.6	60.8	42.0	75.0
Divorced, widowed, separated	48.1	31.5	37.7	24.8	19.4	22.2	42.7	30.0	44.9	19.3
Never married	6.3	7.5	16.5	4.8	0	4.8	7.7	9.3	13.0	5.7
Education										
No degree	24.6	5.6	72.5	62.7	47.2	36.2	34.8	16.7	19.0	6.3
High school diploma or equivalent	57.3	48.2	21.3	25.5	44.4	40.7	44.9	46.7	27.4	29.7
Some college or beyond	18.2	46.2	6.1	11.7	8.4	23.1	20.3	36.7	53.6	64.0
Years in United States										
Born in United States	97.3	96.1	4.8	5.3	0	2.3	53.6	51.3	40.7	20.4
<15 years	0.1	0.2	18.2	14.1	46.1	29.6	6.7	2.0	18.7	5.3
⩾15 years	2.6	3.7	77.0	80.6	53.9	68.0	39.8	46.7	40.6	74.3
Family income as percentage of poverty line										
Poor/negative	16.8	7.5	28.1	22.2	22.1	22.8	27.2	9.6	9.7	6.7
Near poor	7.0	3.5	5.8	6.1	12.9	13.7	2.5	5.2	9.3	2.5
Low income	22.3	10.3	26.4	21.3	11.5	15.9	21.2	13.0	14.5	7.0
Middle income	29.3	25.5	24.0	35.6	39.4	21.6	26.4	30.5	11.4	24.3
High income	24.6	53.2	15.7	14.8	14.1	25.9	22.7	41.7	55.1	59.5
Insurance coverage										
Private	45.2	73.0	11.9	37.9	3.2	34.0	42.6	62.6	50.8	75.6
Public	51.8	23.8	78.3	40.9	94.0	60.3	55.9	29.3	38.7	20.4
Uninsured	3.0	3.2	9.8	21.2	2.7	5.7	1.5	8.1	10.6	4.0
Dental insurance	15.9	39.8	3.4	21.0	0	16.3	14.6	42.3	28.8	50.6
Dental visit in the last year	15.8	55.3	7.4	21.3	14.7	27.6	18.6	38.1	24.3	49.4
Smoker within last year	18.4	7.6	2.0	4.0	0	5.4	9.6	5.1	1.7	2.7

LEP: limited English proficiency.

Table 3 shows that relative to English only participants, those proficient in English, but who spoke languages other than English were less likely to have complete tooth loss (aOR Spanish EP: 0.49 (95% CI: 0.35–0.68); aOR Other Language, EP: 0.62 (95% CI: 0.38–1.02)). These estimates did not vary substantially after adjustment for education and income. Spanish speaking older adults with LEP had a 23% excess odds of complete tooth loss relative to those who communicate in English only (95% CI: 0.92–1.63). Additional adjustment for education revealed a “reversal of the odds” with complete tooth loss less common among those with Spanish LEP relative to English only (aOR: 0.56; 95% CI: 0.42–0.76).

**Table 3.** Association between English language proficiency (LEP) and complete tooth loss among adults aged ⩾50 years in the United States (2017).

	Spanish, LEP	Other language, LEP	Spanish, EP	Other, EP	English only
% with complete tooth loss	13.7	16.9	5.0	6.0	12.0
Crude OR (95% CI)	1.17 (0.88–1.55)	1.49 (0.81–2.74)	0.38 (0.27–0.54)	0.46 (0.28–0.75)	1.0
Partially adjusted OR (95% CI) (age, sex, marital status, smoking status, and dental insurance)	1.23 (0.92–1.63)	1.26 (0.70–2.26)	0.49 (0.35–0.68)	0.62 (0.38–1.02)	1.0
Partially adjusted OR (95% CI) adding *family income*	0.95 (0.71–1.28)	0.94 (0.49–1.79)	0.44 (0.32–0.62)	0.61 (0.36–1.01)	1.0
Partially adjusted OR (95% CI) adding *education*	0.56 (0.42–0.76)	0.80 (0.42–1.53)	0.37 (0.27–0.53)	0.68 (0.40–1.15)	1.0
Partially adjusted OR (95% CI) adding *family income and education*	0.53 (0.39-0.71)	0.72 (0.37-1.39)	0.37 (0.26-0.52)	0.67 (0.39-1.14)	1.0

LEP: limited English proficiency; OR: odd ratio; CI: confidence interval; EP: English proficiency.

**Figure 2.**
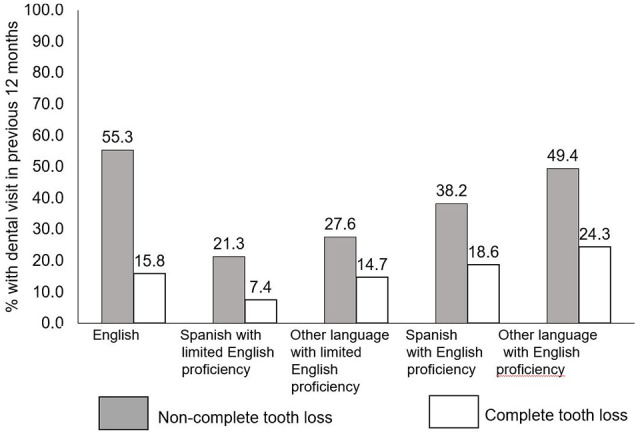
Percent with dental visit in previous 12 months by English language proficiency and edentulism status among adults aged ⩾50 years in the United States (2017).

Figure 2 shows the proportion of adults ⩾50 years of age who reported a dental visit in the 12 months before their interview by LEP category, stratified by edentulism status. The prevalence of dental visits in the past 12 months was higher among people without complete tooth loss relative to those with complete tooth loss, regardless of LEP category. Those with LEP were less likely to report a dental visit in the past year (Spanish: 7.4% with and 21.3% without complete tooth loss; other: 14.7% with and 27.6% without complete tooth loss).

Table 4 shows that among those without complete tooth loss, all LEP categories were less likely to report a dental visit in the past year relative to English only participants. Adjustment for age, sex, marital status, smoking, and dental insurance did not materially alter these estimates, nor did additional adjustment for family income or education. Participants with LEP (Spanish: aOR (0.47; 95% CI: 0.36–0.62); other language: aOR (0.50; 95% CI: 0.29–0.86) had half the odds of reporting a dental visit in the year previous relative to English only participants. Participants proficient in English (Spanish: aOR (0.60; 95% CI: 0.49–0.74). Other language: aOR (0.68; 95% CI: 0.52–0.88) had reduced odds of reporting a dental visit in the year previous relative to English only participants. Among those without complete tooth loss, the 95% CIs demonstrate that the lack of precision necessary to yield informative results.

**Table 4.** Association between English language proficiency (LEP) and dental visit in past 12 months, stratified by complete tooth loss among adults aged ⩾50 years in the United States (2017).

	Spanish, LEP	Other language, LEP	Spanish, EP	Other, EP	English only
	Among those without complete tooth loss (weighted n = 99,161,606)				
Crude OR (95% CI)	0.23 (1.17–0.29)	0.31 (0.18–0.52)	0.50 (0.41–0.60)	0.79 (0.63–1.00)	1.0
Partially adjusted OR (95% CI) (age, sex, marital status, smoking status, and dental insurance)	0.24 (0.18–0.32)	0.31 (0.18–0.54)	0.51 (0.42–0.63)	0.73 (0.57–0.94)	1.0
Partially adjusted OR (95% CI) adding *family income*	0.31 (0.24–0.41)	0.41 (0.24–0.70)	0.55 (0.43–0.67)	0.73 (0.57–0.94)	1.0
Partially adjusted OR (95% CI) adding *education*	0.42 (0.32–0.55)	0.41 (0.24–0.73)	0.58 (0.48–0.71)	0.66 (0.51–0.87)	1.0
Partially adjusted OR (95% CI) adding *family income and education*	0.47 (0.36–0.62)	0.50 (0.29–0.86)	0.60 (0.49–0.74)	0.68 (0.52–0.88)	1.0
	Among those with complete tooth loss (weighted n = 12,733,684)				
Crude OR (95% CI)	0.43 (0.17–1.07)	0.92 (0.37–2.30)	1.22 (0.56–2.65)	1.71 (0.41–7.15)	1.0
Partially adjusted OR (95% CI) (age, sex, marital status, smoking status, and dental insurance)	0.49 (0.19–1.26)	1.29 (0.51–3.26)	1.24 (0.56–2.77)	1.33 (0.43–4.15)	1.0
Partially adjusted OR (95% CI) adding *family income*	0.50 (0.20–1.28)	1.37 (0.53–3.52)	1.29 (0.57–2.91)	1.22 (0.39–3.82)	1.0
Partially adjusted OR (95% CI) adding *education*	0.66 (0.25–1.73)	1.50 (0.58–3.84)	1.32 (0.60–2.91)	1.11 (0.36–3.38)	1.0
Partially adjusted OR (95% CI) adding *family income and education*	0.66 (0.25–1.73)	1.55 (0.61–3.98)	1.35 (0.61–2.99)	1.06 (0.34–3.28)	1.0

LEP: limited English proficiency; OR: odds ratio; CI: confidence interval; EP: English proficiency.

## Discussion

In the United States, the population is aging and becoming more diverse, such that the proportion of people with LEP is growing rapidly. This study sought to provide population-based estimates of complete tooth loss by LEP status and to estimate the proportion of older adults in the United States who had a recent dental visit by LEP status. We found that complete tooth loss varied by English language proficiency among adults aged ⩾50 years in the United States. Analyses adjusted for a variety of factors induced a reversal of the odds with reduced odds of complete tooth loss among those who spoke languages other than English, relative to those who reported English only. We also found that the proportion of people reporting a dental visit in the past 12 months was suboptimal and varied by LEP and whether people had complete tooth loss. These findings are important for dental health services planning given the increased diversity among an aging population in the United States.

In 2017, 11.4% of non-institutionalized, civilian persons in the United States aged ⩾50 years reported complete tooth loss. We found that older adults with complete tooth loss were more likely to have public insurance as compared to those without complete tooth loss. In the United States, public insurance is available through a joint federal and state program called Medicaid to people who have low-income or who qualify for public insurance based on certain disabilities or pregnancy or through Medicare which is available to all citizens aged ⩾65 years. We found that the prevalence of complete tooth loss varied across English language proficiency groups. Relative to adults aged ⩾50 years who only spoke English, those who were proficient in English yet spoke another language at home were less likely to report complete tooth loss. This may reflect different in socioeconomic positioning by language proficiency. Conversely, non-English speakers who were not proficient in English were more likely to report complete tooth loss. Interestingly, English-only populations had the highest odds of experiencing complete tooth loss compared to different language populations after adjustment for potential confounders. We did not have information on dental insurance or other factors that may explain oral health across the lifespan. Distal factors (e.g. access to dental care in childhood, nutrition in childhood) may be important drivers of complete tooth loss in older age that must be considered in future research. These intriguing findings may be viewed as consistent with a large, cross-national study which substantiated the association between socioeconomic conditions in the early years of life and tooth retention.^15^ We viewed educational attainment as a marker for socioeconomic positioning in earlier life. Adjustment for this variable reversed the estimates of the aORs. The cross-sectional nature of the MEPS data impeded our ability to disentangle these intriguing findings further.

We found that many older adults in the United States did not have an annual dental visit. Older adults with LEP were the least likely to report having a dental visit in the previous year. This is consistent with previous research. For example, among older adults ⩾65 years of age in the United States, 34.4% of Latinos had untreated dental caries (compared to 21.8% of non-Hispanic Whites), and many did not have a dental visit in the past year.^16,17^ Cost was noted as a barrier in fewer than 10%.^16^ Ethnic minorities were at greater risk for cost-related delayed or foregone dental care.^18^

We also found that the differences in dental visits in the past year varied by edentulism status. People with complete tooth loss were the least likely to report having seen a dental care provider in the last year regardless of language proficiency. The American College of Prosthodontists recommend that persons with complete tooth loss visit a dental care provider annually to evaluate their oral health, because biological changes to the soft and hard tissues of the mouth can alter how dentures fit. The American College of Prosthodontists official statement on dentures highlights that persons with ill-fitting dentures have increased risk of developing head and neck cancer of other carcinogenic contributors are present.^19,20^ Ill-fitting dentures effects food maceration and social acceptance.^21,22^ Dental providers recommend that persons who use complete dentures or artificial implants routinely visit their dental care provider to prevent ill-fitting dentures, and other potential oral health concerns.^20,22^ While we cannot estimate how many persons with complete tooth loss have the necessary dental prosthetics for a complete dentition, we can say that the population with complete tooth loss is not frequenting dental providers at rates effective for oral health maintenance.

### Strengths and Limitations

This study provides contemporaneous, population-based estimates of complete tooth loss across categories by English language proficiency. Interviewers were fully functional in multiple languages.^11,13^ Our study applied robust statistical techniques that allowed us to estimate population-level prevalence of complete tooth loss and recent dental visits among vulnerable populations. Our sample size permitted us to differentiate Spanish speaking older adults from other languages. The United States has the second highest number of Spanish speaking people in the world.^23^ However, we were unable to further categorize other languages. Despite these strengths, the study does have some limitations to keep in mind. The two primary outcome variables were self-reported.^24^ However, self-reported complete tooth loss has been shown to be valid.^25,26^ Power calculations were not performed. We recognize that estimates of recent dental visits among older adults with complete tooth loss lack precision. Furthermore, we were unable to evaluate other forms of dental health care utilization (e.g. use of surgical implants, repairing dentures) because few people in the study reported use of these dental services.

## Conclusion

The goals of this study were to calculate population-based estimates of complete tooth loss by LEP status among older adults in the United States and to estimate the proportion of older adults in the United States who had a recent dental visit by LEP status. In 2017, 11.4% of the United States population aged ⩾50 years had complete tooth loss. While overall adherence to recommended annual visits with oral health providers was suboptimal, striking disparities among those with LEP were observed and may have been exacerbated by complete tooth loss. Among those without complete tooth loss, differences in adherence to annual dental visits across LEP categories were not explained by dental insurance, suggesting that further study of the role of additional barriers such as access to dental clinics, education regarding the importance of regular routine dental care, and dental provider preparedness to meet the care needs of diverse patients is warranted. Further research is sorely needed in this area to meet the needs of older adults as our aging population diversifies.

## Supplemental Material

2017_SAQ_ENG – Supplemental material for English language proficiency, complete tooth loss, and recent dental visits among older adults in the United States Click here for additional data file. Supplemental material, 2017_SAQ_ENG for English language proficiency, complete tooth loss, and recent dental visits among older adults in the United States by Andriana M Foiles Sifuentes, Maira A Castaneda-Avila and Kate L Lapane in SAGE Open Medicine
