# Supplementary material for: English language proficiency, complete tooth loss, and recent dental visits among older adults in the United States
Source: SAGE Open Med. 2020 Oct 7;8:2050312120962995. doi: 10.1177/2050312120962995 (PMC7550941; doi:10.1177/2050312120962995)
Supplement: 2017_SAQ_ENG – Supplemental material for English language proficiency, complete tooth loss, and recent dental visits among older adults in the United States [file 2017_SAQ_ENG.pdf]

3490

Form Approved
OMB# 0935-0118
Exp. Date 12/31/2018

2017

Your Health and Health Opinions

Your opinion matters!

MEPS
Medical Expenditure Panel Survey

Understanding how people feel about their health and health care is an important goal of MEPS. Please take a few minutes to answer the questions in this booklet.

Survey Instructions

- Please answer every question by marking one box "☒." If you are unsure about how to answer a question, please give the best answer you can.
- You are sometimes told to skip over some questions in this survey. When this happens you will see arrows that tell you what questions to answer next, like this:

  ☐ Yes
  ☐ No ➔ **If No, go to 3**

  Next Question

- Your participation is voluntary and all of your answers will be kept confidential to the extent permitted by law. If you have any questions about this booklet, please call Alex Scott at 1-800-945-MEPS (6377).
- Store your completed booklet in the envelope provided. Have it ready to give to your interviewer at his or her next visit.

This Booklet Should Be Completed By ➔

REGION: ☐ **RUID:** ☐☐☐☐☐☐☐ **PID:** ☐☐☐

NAME: ______________________________

____

DOB: ☐☐ / ☐☐ / ☐☐☐☐
MONTH DAY YEAR

This survey is authorized under 42 U.S.C. 299a. The confidentiality of your responses to this survey is protected by Sections 944(c) and 308(d) of the Public Health Service Act [42 U.S.C. 299c-3(c) and 42 U.S.C. 242m(d)]. Information that could identify you will not be disclosed unless you have consented to that disclosure. Public reporting burden for this collection of information is estimated to average 7 minutes per response, the estimated time required to complete the survey. An agency may not conduct or sponsor, and a person is not required to respond to, a collection of information unless it displays a currently valid OMB control number. Send comments regarding this burden estimate or any other aspect of this collection of information, including suggestions for reducing this burden, to: AHRQ Reports Clearance Officer Attention: PRA, Paperwork Reduction Project (0935-0118) AHRQ, 5600 Fishers Lane, Room #07W42, Rockville, MD 20857.

DEPARTMENT OF HEALTH & HUMAN SERVICES • USA

The Agency for Healthcare Research and Quality and
The Centers for Disease Control and Prevention of the
U.S. Department of Health and Human Services

General Health

1. In general, would you say your health is:

- ☐ Excellent
- ☐ Very good
- ☐ Good
- ☐ Fair
- ☐ Poor

2. The following items are about activities you might do during a typical day. Does **your health <u>now</u> limit you** in these activities? If so, how much?

a. **Moderate activities,** such as moving a table, pushing a vacuum cleaner, bowling, or playing golf

- ☐ Yes, limited a lot
- ☐ Yes, limited a little
- ☐ No, not limited at all

b. Climbing **several** flights of stairs

- ☐ Yes, limited a lot
- ☐ Yes, limited a little
- ☐ No, not limited at all

3. During the **<u>past 4 weeks,</u>** have you had any of the following problems with your work or other regular daily activities **as a result of your physical health?**

a. **Accomplished less** than you would like **as a result of your physical health**

- ☐ No, none of the time
- ☐ Yes, a little of the time
- ☐ Yes, some of the time
- ☐ Yes, most of the time
- ☐ Yes, all of the time

b. Were limited in the **kind** of work or other activities **as a result of your physical health**

- ☐ No, none of the time
- ☐ Yes, a little of the time
- ☐ Yes, some of the time
- ☐ Yes, most of the time
- ☐ Yes, all of the time

"VR-12: How to create VR-12 scales and PCS/MCS summaries" © 2014 by Trustees of Boston University. All Rights Reserved.

(Questions concerning the VR-12 can be directed to Professor Lewis E. Kazis, Boston University e-mail: lek@bu.edu)

3490

4. During the **past 4 weeks,** have you had any of the following problems with your work or other regular daily activities **as a result of any emotional problems** (such as feeling depressed or anxious)?

   a. **Accomplished less** than you would like **as a result of any emotional problems**

   - ☐ No, none of the time
   - ☐ Yes, a little of the time
   - ☐ Yes, some of the time
   - ☐ Yes, most of the time
   - ☐ Yes, all of the time

   b. Didn't do work or other activities as **carefully** as usual **as a result of any emotional problems**

   - ☐ No, none of the time
   - ☐ Yes, a little of the time
   - ☐ Yes, some of the time
   - ☐ Yes, most of the time
   - ☐ Yes, all of the time

5. During the **past 4 weeks,** how much did **pain** interfere with your normal work (including both work outside the home and housework)?

   - ☐ Not at all
   - ☐ A little bit
   - ☐ Moderately
   - ☐ Quite a bit
   - ☐ Extremely

3490

These questions are about how you feel and how things have been with you during the **past 4 weeks.** For each question, please give the one answer that comes closest to the way you have been feeling.

6. How much of the time during the **past 4 weeks:**

a. Have you felt calm and peaceful?

- ☐ All of the time
- ☐ Most of the time
- ☐ A good bit of the time
- ☐ Some of the time
- ☐ A little of the time
- ☐ None of the time

b. Did you have a lot of energy?

- ☐ All of the time
- ☐ Most of the time
- ☐ A good bit of the time
- ☐ Some of the time
- ☐ A little of the time
- ☐ None of the time

c. Have you felt downhearted and blue?

- ☐ All of the time
- ☐ Most of the time
- ☐ A good bit of the time
- ☐ Some of the time
- ☐ A little of the time
- ☐ None of the time

7. During the **past 4 weeks,** how much of the time has your **physical health or emotional problems** interfered with your social activities (like visiting with friends, relatives, etc.)?

- ☐ All of the time
- ☐ Most of the time
- ☐ Some of the time
- ☐ A little of the time
- ☐ None of the time

8. The following questions ask about how you have been feeling during **the past 30 days.** For each question, please mark the box that best describes how often you had this feeling.

During the past 30 days, about how often did you feel...	**All of the time**	**Most of the time**	**Some of the time**	**A little of the time**	**None of the time**
a. nervous?	☐	☐	☐	☐	☐
b. hopeless?	☐	☐	☐	☐	☐
c. restless or fidgety?	☐	☐	☐	☐	☐
d. so sad that nothing could cheer you up?	☐	☐	☐	☐	☐
e. that everything was an effort?	☐	☐	☐	☐	☐
f. worthless?	☐	☐	☐	☐	☐

9. The following two questions ask about how you have been feeling in the **past 2 weeks.**

Over the last 2 weeks, how often have you been bothered by any of the following problems?	**Nearly every day**	**More than half the days**	**Several days**	**Not at all**
a. Little interest or pleasure in doing things	☐	☐	☐	☐
b. Feeling down, depressed, or hopeless	☐	☐	☐	☐

3490

Opinions About Health

10. For the four statements below, please mark **one** of the boxes to indicate how strongly you **agree** or **disagree** for each statement. If you are uncertain, mark the box for uncertain.

	Disagree strongly	Disagree somewhat	Uncertain	Agree somewhat	Agree strongly
a. I'm healthy enough that I really don't need health insurance........................	☐	☐	☐	☐	☐
b. Health insurance is not worth the money it costs....................................	☐	☐	☐	☐	☐
c. I'm more likely to take risks than the average person..................................	☐	☐	☐	☐	☐
d. I can overcome illness without help from a medically trained person..........	☐	☐	☐	☐	☐

Your Health Care in the Last 12 Months

These questions ask about your own health care. Do **not** include care you got when you stayed overnight in a hospital. Do **not** include the times you went for dental care visits.

11. In the last 12 months, did you have an illness, injury, or condition **that needed care right away** in a clinic, emergency room, or doctor's office?

- ☐ Yes
- ☐ No → **If No, go to 13**

12. In the last 12 months, when you **needed care right away,** how often did you get care as soon as you thought you needed?

- ☐ Never
- ☐ Sometimes
- ☐ Usually
- ☐ Always

13. In the last 12 months, did you make any appointments for a **check-up or routine care** at a doctor's office or clinic?

- ☐ Yes
- ☐ No → **If No, go to 15**

If Yes, go to 14

3490

14. In the last 12 months, how often did you get an appointment for a **check-up or routine care** at a doctor's office or clinic as soon as you needed?

- ☐ Never
- ☐ Sometimes
- ☐ Usually
- ☐ Always

15. In the last 12 months, **not** counting the times you went to an emergency room, how many times did you go to a doctor's office or clinic to get health care for yourself?

- ☐ None ➔ **If None, go to 26**
- ☐ 1 time
- ☐ 2
- ☐ 3
- ☐ 4
- ☐ 5-9
- ☐ 10 or more times

16. Using any number from 0 to 10, where 0 is the worst health care possible and 10 is the best health care possible, what number would you use to rate all your health care in the last 12 months?

- ☐ 0 Worst health care possible
- ☐ 1
- ☐ 2
- ☐ 3
- ☐ 4
- ☐ 5
- ☐ 6
- ☐ 7
- ☐ 8
- ☐ 9
- ☐ 10 Best health care possible

3490

17. In the last 12 months, did a doctor or other health provider give you instructions about what to do about a specific illness or health condition?

- ☐ Yes
- ☐ No ➔ **If No, go to 20**

18. In the last 12 months, how often were these instructions easy to understand?

- ☐ Never
- ☐ Sometimes
- ☐ Usually
- ☐ Always

19. In the last 12 months, how often did doctors or other health providers ask you to describe how you were going to follow these instructions?

- ☐ Never
- ☐ Sometimes
- ☐ Usually
- ☐ Always

20. In the last 12 months, did you have to fill out or sign any forms at a doctor's or other health provider's office?

- ☐ Yes
- ☐ No ➔ **If No, go to 22**

21. In the last 12 months, how often were you offered help in filling out a form at the doctor's or other health provider's office?

- ☐ Never
- ☐ Sometimes
- ☐ Usually
- ☐ Always

3490

22. In the last 12 months, how often did doctors or other health professionals explain things in a way that was easy to understand?

- ☐ Never
- ☐ Sometimes
- ☐ Usually
- ☐ Always

23. In the last 12 months, how often did doctors or other health professionals listen carefully to you?

- ☐ Never
- ☐ Sometimes
- ☐ Usually
- ☐ Always

24. In the last 12 months, how often did doctors or other health professionals show respect for what you had to say?

- ☐ Never
- ☐ Sometimes
- ☐ Usually
- ☐ Always

25. In the last 12 months, how often did doctors or other health professionals spend enough time with you?

- ☐ Never
- ☐ Sometimes
- ☐ Usually
- ☐ Always

26. Do you currently smoke?

- ☐ Yes
- ☐ No ➔ **If No, go to the top of the next page**

27. In the last 12 months, did a doctor advise you to quit smoking?

- ☐ Yes
- ☐ No
- ☐ Had no visits in the last 12 months

3490

Getting Health Care from Specialists

When you answer the next questions, do **not** include dental visits or care you got when you stayed overnight in a hospital.

28. Specialists are doctors like surgeons, heart doctors, allergy doctors, skin doctors, and other doctors who specialize in one area of health care. In the last 12 months, did you make any appointments to see a specialist?

- ☐ Yes
- ☐ No ➔ **If No, please go to the "Date completed" boxes below**

29. In the last 12 months, how often did you get an appointment to see a specialist as soon as you needed?

- ☐ Never
- ☐ Sometimes
- ☐ Usually
- ☐ Always

Date completed: ☐☐ Month ☐☐ Day ☐☐☐☐ Year

Who completed this form?

- ☐ Person named on front of this form
- ☐ Someone else,

If Someone Else, what is person's relationship to the person named on the front of this form?

- ☐ Husband or wife
- ☐ Unmarried partner
- ☐ Mother, father, or guardian
- ☐ Son or daughter
- ☐ Other relative
- ☐ Not related

Thank you for taking the time to complete this survey.

Remember to store it in the envelope provided.
